# Limited evidence of biased offspring sex allocation in a cavity-nesting conspecific brood parasite

**DOI:** 10.1093/beheco/arae024

**Published:** 2024-03-29

**Authors:** Caitlin P Wells, Bruce E Lyon, Caroline M Thow, Tez Stair, Melissa Jones, Mitch Hinton, John M Eadie

**Affiliations:** Department of Fish, Wildlife, and Conservation Biology; Colorado State University, 901 Amy Van Dyken Way, Fort Collins, CO 80523, USA; Department of Wildlife, Fish, and Conservation Biology; University of California, 1088 Academic Surge, Davis, CA 95616, USA; Department of Ecology and Evolution; University of California, UCSC/Coastal Biology Building 130 McAllister Way, Santa Cruz, CA 95060, USA; Department of Ecology and Evolution; University of California, UCSC/Coastal Biology Building 130 McAllister Way, Santa Cruz, CA 95060, USA; Department of Wildlife, Fish, and Conservation Biology; University of California, 1088 Academic Surge, Davis, CA 95616, USA; Department of Wildlife, Fish, and Conservation Biology; University of California, 1088 Academic Surge, Davis, CA 95616, USA; Department of Wildlife, Fish, and Conservation Biology; University of California, 1088 Academic Surge, Davis, CA 95616, USA; Department of Wildlife, Fish, and Conservation Biology; University of California, 1088 Academic Surge, Davis, CA 95616, USA

**Keywords:** alternative reproductive tactic, density, female philopatry, local resource competition, local resource enhancement, nest box study, offspring sex ratio, waterfowl

## Abstract

Sex allocation theory predicts that mothers should bias investment in offspring toward the sex that yields higher fitness returns; one such bias may be a skewed offspring sex ratio. Sex allocation is well-studied in birds with cooperative breeding systems, with theory on local resource enhancement and production of helpers at the nest, but little theoretical or empirical work has focused on birds with brood parasitic breeding systems. Wood ducks (*Aix sponsa*) are a conspecific brood parasite, and rates of parasitism appear to increase with density. Because female wood ducks show high natal philopatry and nest sites are often limiting, local resource competition (LRC) theory predicts that females should overproduce male offspring—the dispersing sex—when competition (density) is high. However, the unique features of conspecific brood parasitism generate alternative predictions from other sex allocation theory, which we develop and test here. We experimentally manipulated nesting density of female wood ducks in 4 populations from 2013 to 2016, and analyzed the resulting sex allocation of >2000 ducklings. In contrast to predictions we did not find overproduction of male offspring by females in high-density populations, females in better condition, or parasitic females; modest support for LRC was found in overproduction of only female parasitic offspring with higher nest box availability. The lack of evidence for sex ratio biases, as expected for LRC and some aspects of brood parasitism, could reflect conflicting selection pressures from nest competition and brood parasitism, or that mechanisms of adaptive sex ratio bias are not possible.

## Teaser

If you might compete with your daughters for limited nest sites, should you produce sons instead? Many female mammals do, but female wood ducks apparently don’t. As conspecific brood parasites that lay eggs in their own and others’ nests, female wood ducks might experience mother-daughter competition differently: even if many nests are occupied, daughters might be able to lay eggs in mothers’ and others’ nests as parasites, so mothers don’t shift to sons.

## Introduction

Understanding offspring sex allocation remains a major goal in evolutionary biology (West 2009). At the population level, Fisher (1930) attempted to explain observations of balanced (50:50) sex ratios with his theory of equal allocation, in which frequency-dependent selection for the rarer sex makes equal investment in male and female offspring an evolutionarily stable strategy. At the individual level, most work on sex allocation in vertebrates has focused on testing the Trivers and Willard (1973) model of sex ratio adjustment (Silk and Brown 2008). According to this model, females bias offspring sex according to their own heritable body condition: higher quality females toward the sex that will disproportionately benefit from higher body condition, and lower quality females toward the sex that will suffer less from poorer body condition (Trivers and Willard 1973). The traditional Trivers–Willard model predicts that for highly polygynous species, in which male offspring have a higher variance in reproductive success than female offspring, mothers in good condition should produce more sons (Trivers and Willard 1973). Conversely, the “reverse” Trivers–Willard model predicts that if females show higher variance in reproductive success than males—e.g. sex-role reversed species (Andersson et al. 2003) or species with intense female-female competition—mothers in good condition should produce more daughters (Schindler et al. 2015). A smaller set of studies addresses sex allocation according to resource availability, where females bias offspring sex toward the dispersing sex when resources are scarce and relatives compete for those resources (Local Resource Competition, LRC, Clark 1978; Silk 1983), or toward the philopatric sex when those offspring help rear additional siblings (Local Resource Enhancement, LRE Gowaty and Lennartz 1985; Emlen et al. 1986). However, these theories are not mutually exclusive, and the operation of multiple, often opposing, selection pressures renders predictions about the direction of offspring sex ratio bias difficult (Wild and West 2007; Schindler et al. 2015).

Birds have been the focus of considerable sex ratio investigations, including some testing LRC. Evidence in support of LRC comes mainly from secondary cavity-nesting species, as access to cavities strongly limits breeding opportunities and should lead to competition (Song et al. 2016). In these species, primary sex ratios have been biased toward the philopatric sex when resources were abundant, as measured either from decreasing population size (Hjernquist et al. 2009), higher nest-box abundance (Song et al. 2016), or lower density of breeding conspecifics (Michler et al. 2013). However, all of these tests were conducted in species with male natal philopatry (great tits (*Parus major*), Michler et al. 2013; Song et al. 2016; collared flycatchers (*Ficedula albicollis*), Hjernquist et al. 2009). In cavity-nesting species with female natal philopatry, namely cavity-nesting waterfowl, female–female competition for limited nest sites can be intense (Semel and Sherman 2001; Harvey et al. 2021) and LRC might be expected. Yet, despite expectations that LRC or LRE could select for biased offspring sex ratios in female-philopatric waterfowl, studies are rare (Jaatinen et al. 2013).

Differential sex allocation of offspring could also be associated with alternative reproductive tactics (ARTs). In males, ARTs typically involve different tactics to obtain matings (Oliveira et al. 2008). Theory suggests that different alternative tactics might be associated with different sex allocation patterns (Alonzo and Sinervo 2007) and such patterns have been documented in some species (Shuster and Sassaman 1997). Females also adopt ARTs in some species but these center on patterns of egg laying and parental care (e.g., Sinervo et al. 2000; Ferrari et al. 2019). Conspecific brood parasitism, whereby females lay eggs in the nests of other conspecifics, is an ART that is widespread in birds (Yom-Tov 2001; Lyon and Eadie 2008). In these species females may follow one of several tactics: lay eggs in and exclusively incubate one’s own nest (Nesting female), lay eggs exclusively in nests incubated by other females (Parasite female), or lay eggs in one’s own and other nests (Nesting Parasite) (Lyon and Eadie 2008). Conspecific brood parasitism is common in cavity-nesting waterfowl, and nesting females may experience additional competition or cooperation from unrelated or related parasitic females (Lyon and Eadie 2008; Andersson et al. 2015).

We studied sex allocation in the wood ducks (*Aix sponsa*), a cavity-nesting species of waterfowl that breeds widely across North America (Bellrose and Holm 1994). As in other waterfowl females are the philopatric sex (Hepp et al. 1989). Females compete for nest sites, which can be a limiting resource (Semel and Sherman 2001), and hence should experience increased local resource competition when conspecific densities are high or nest sites are scarce. Additionally, conspecific brood parasitism is common, and within a year all 3 possible reproductive strategies occur (see above for definitions; Thow 2019). Some sex ratio predictions may be specific to particular reproductive strategies.

We test predictions of sex ratio bias—at the individual and population level—according to multiple theories of sex allocation. First, we experimentally manipulated the nesting density of wood ducks in 4 populations to examine the effect of local resource competition on offspring sex allocation in a cavity-nesting species with female philopatry. Additionally, recognizing that larger body size or condition may influence the success of females in competition for limited nest sites, we ask if females in better body condition produce male-biased or female-biased offspring sex ratios, according to traditional or “reverse” Trivers-Willard expectations, respectively. Last, we ask if females pursuing a parasitic ART (i.e. parasite or nesting parasite) produce either male- or female-biased offspring sex ratios in this species of conspecific brood parasite.

The unique features of conspecific brood parasitism bring considerable richness to sex allocation theories, and it is not immediately clear what predictions to make. The predicted outcome will depend on several aspects of a species’ biology, which are currently unknown for wood ducks: does maternal condition disproportionately advantage male versus female offspring, even when nest competition is strong? In species with female philopatry, does kinship come into play and reduce or intensify the costs of parasitism? Previous studies have not explored the expectations of sex allocation theory for a brood parasitic system and we do so here in the *Conceptual Framework* below.

## Conceptual framework

Our framework provides predictions for alternative mechanisms and hypotheses that could apply to our system with competition for limited resources (nest sites) among female relatives and social competition through conspecific brood parasitism: local resource competition, local resource enhancement, Trivers–Willard effect (including alternative reproductive tactics (ARTs), supernumerary egg, and laying sequence patterns), and reverse Trivers-Willard effect.

### Local resource competition

When female relatives compete for limiting resources (local resource competition), male-biased sex ratios may be favored. Clark’s (1978) local resource competition theory focused specifically on competition among close female relatives in small groups of kin-associated species like primates. Silk (1983, 1984) expanded this idea to apply to larger groups containing both kin and unrelated individuals and confirmed the generality of male-based sex ratios under local resource competition. Female cavity nesting waterfowl might be expected to show local resource competition due to strong female natal philopatry and competition for limited nest sites. Females also show reproductive competition through brood parasitism. If parasitism is both costly and sometimes targets relatives, the conditions for resource competition would be met. Therefore both nest site competition and parasitism could favor individual and population male-biased sex ratios, particularly at high densities where such competition will be most intense.

### Local resource enhancement

In some situations, sex-specific kin interactions may be beneficial and favor biased sex ratios towards the beneficial sex. This local resource enhancement effect has been proposed for species with helpers at the nest in birds (Gowaty and Lennartz 1985). A similar effect could apply to kin-biased brood parasitism, but requires quite specific assumptions about the context of brood parasitism. Andersson (2001, 2017) suggested that female waterfowl with nests could enhance the reproductive success of female relatives by allowing them to lay parasitic eggs in their nest. Such kin-facilitated parasitism is advantageous to hosts when it increases either the reproduction (via hatching of parasitic eggs) or survival (via reduction in predation risk by not nesting) of relatives, and therefore predicts the highest inclusive fitness gains from non-nesting female parasites who lay in the nests of female relatives (Lyon and Eadie 2000; Andersson 2001, 2017; Eadie and Lyon 2011; Andersson et al. 2019). Alternatively, if parasitism is costly to the host, non-nesting parasitic females could increase their relatives’ inclusive fitness by laying in the nests of nonrelatives when those nests are abundant (Lyon and Eadie 2000; Eadie and Lyon 2011). Hence under high density of nesting females, female-biased sex ratios may be favored at both the individual and population level, although the strength of selection favoring such bias would depend on the fraction of females that are able to increase their inclusive fitness by facilitating parasitism by female relatives.

### Trivers–Willard

Body size or condition of female waterfowl could confer an advantage to sons in competing for female mates and hence are reasonable to examine under traditional Trivers–Willard expectations. Additionally, body condition is thought to affect female ART in conspecific brood parasites, with females in better condition able to use a nesting parasitic strategy (Sorenson 1991; Lyon and Eadie 2008): Nesting parasite females are distinct from both nesting and parasite females in that they lay supernumerary eggs (i.e., eggs laid beyond the usual clutch size), allowing them to increase and even double their reproductive success (Lyon 1993, Åhlund and Andersson 2001). In general, avian mothers with a higher capacity for investment can produce more supernumerary eggs than mothers with a lower capacity for investment (Nager et al. 1999, 2000; Bowers et al. 2014). Sex ratio has been observed to vary across the laying sequence (Cassey et al. 2006), often in line with Trivers–Willard expectations: because egg production is costly, females are in better condition at the beginning of the laying sequence, and egg sex ratios shift from male-biased early in the laying sequence to female-biased late in the laying sequence as female condition declines (e.g., Ankney 1982; Nager et al. 1999; Krebs et al. 2002; Velando et al. 2002; Vedder et al. 2013). However, mothers able to produce more supernumerary eggs have also produced male-biased clutches (Bowers et al. 2014). Collectively, these studies show that sex allocation can change across the laying order and that those changes are particularly pronounced in clutches with supernumerary eggs. In our populations of wood ducks, nesting parasites lay the largest number of eggs and produce more ducklings than nesting females and nearly double that of parasite females (Thow 2019). Across species, nesting parasites typically lay parasitic eggs before nest eggs (Lyon 1993, Andersson and Åhlund 2012), and in wood ducks 96% of nesting parasites have been observed to do so (Semel and Sherman 2001); hence, nest eggs include supernumerary eggs. To allow for sex allocation to change across laying order, we consider parasitic versus nest eggs separately for nesting parasites. Following traditional Trivers–Willard logic we would expect that nesting parasite ducklings should be male-biased, or that early-laid parasitic ducklings may be male-biased but nest ducklings could be at parity or female-biased (Trivers and Willard 1973; Bowers et al. 2014).

### Reverse Trivers–Willard

Many species experience strong social selection due to female competition (Tobias et al. 2012)—if large females or females in better condition are better competitors, such females should bias their offspring sex ratio if size or condition is repeatable across generations (Andersson et al. 2003; Schindler et al. 2015). A reverse Trivers–Willard effect could be generated by the effects of female quality on social competition over nest sites or on fitness through brood parasitism. Small females or those in poor condition would be expected to bias their offspring towards males, all else equal. For brood parasitism, the context of parasitism is likely important to specific predictions about sex ratio bias because parasite and nesting parasite alternative reproductive tactics likely differ in life-history tradeoffs and the ecological conditions that influence those tradeoffs (Lyon and Eadie 2008). Females pursuing a non-nesting parasite ART are thought to do so because of their lower competitive ability (Best of a Bad Job hypothesis, Lyon and Eadie 2008; Pöysä et al. 2014). Non-nesting parasites have sufficient resources to produce at least one egg, but their own or environmental conditions are not sufficient to enable them to secure their own nest and/or incubate a clutch. Hence if a mother’s competitive ability is correlated with her daughters’, parasite females should produce male-biased offspring sex ratios. In contrast, females pursuing a nesting parasite ART are thought to do so because of their high competitive ability. Nesting Parasites have the resources to not only secure their own nest and incubate a clutch but also to lay supernumerary eggs that increase their reproductive investment and success (fecundity enhancement, Lyon and Eadie 2008). Hence following reverse Trivers–Willard logic we would expect that nesting parasite ducklings should be female-biased, or that early-laid parasitic ducklings may be female-biased but nest ducklings could be at parity or male-biased.

A summary of predictions for each hypothesis at both the population and individual level is provided in Table 1.

**Table 1.** Specific predictions for the effect of individual- and population-level variables on the proportion of male ducklings produced by a species with conspecific brood parasitism, according to multiple hypotheses for sex allocation: LRC (Local Resource Competition, Clark 1978), LRE (Local Resource Enhancement, Gowaty and Lennartz 1985; Emlen et al. 1986), TW (Trivers-Willard, Trivers and Willard 1973), “Reverse” TW (Schindler et al. 2015), and ART (Alternative Reproductive Tactic, this study). Pop = Population, Ind = Individual

Hypothesis	Predictor	Direction of effect (pn male)	Level of effect
LRC	Density: females/ha	+	Pop
	Density: nearest neighbor distance	−	Ind & Pop
	Nest site availability	−	Ind & Pop
	Relatedness	+	Ind & Pop
LRE	Density: females/ha	−	Pop
	Density: nearest neighbor distance	+	Ind & Pop
	Nest site availability	+	Ind & Pop
	Relatedness	−	Ind & Pop
TW	Female size, condition	+	Ind
	ART—parasite	−	Ind
	ART—nesting parasite	+ (or ± by egg type)	Ind
“Reverse” TW	Female size, condition	−	Ind
	ART—parasite	+	Ind
	ART—nesting parasite	− (or ± by egg type)	Ind

## Methods

### Field sites.

We studied Wood Ducks in the Central Valley of California, United States, at 4 sites near the town of Davis, from 2013 to 2016: Russell Ranch (lat 38°32ʹ03″N, long 121°52ʹ05″W, USA), Putah Creek (lat 38°31ʹ0″N, long 121°46ʹ05″W, United States), Conaway Ranch (lat 38°38ʹ24″N, long 121°42ʹ0″W, USA), and Roosevelt Ranch (lat 38°49ʹ15″N, long 121°48ʹ39″W, USA). Sites were remnant oak woodland habitat, persisting in narrow riparian corridors (< 20 m wide on each bank, 2–5 km long) along a stream (Russell Ranch, Putah Creek), slough (Conaway Ranch), or restored and managed wetland complex (Roosevelt Ranch) adjacent to agricultural fields.

### Experimental manipulation.

We capitalized on an ongoing experimental manipulation of nesting female density to examine changes in offspring sex ratio. The availability of natural cavities can range from 1.2 to 1.4 suitable cavities per hectare in northern Minnesota (Beerden et al. 2022), 9.4 cavities/ha in floodplain forest and 14.5 cavities/ha in Illinois upland forest (Nielsen et al. 2007), and up to 15.3 cavity trees/ha in eastern forests (Gilmer et al. 1978). “Natural” nesting densities of wood ducks in natural tree cavities range from 0.05 to 2.87 nests/hectare (Bellrose et al. 1964, Bellrose & Holm 1994, Robb and Bookhout 1995, Yetter et al. 1999). Given that much of the riparian forest has been lost in California (> 95%), we suspect the abundance of natural cavities is lower than in eastern forests. Hence, our manipulated densities of nest boxes from 0.3 boxes/ha to 4.3 boxes/ha and observed female densities from 0.2 to 2.2 females/ha represent a realistic range of nest site availability (from low to high) and created biologically meaningful differences in females/nest density.

The nest manipulation experiment was conducted in several phases. Twenty-eight boxes were present at Conaway Ranch in 1999, but we added additional boxes in 1998 and in 2012 resulting in a total of 72 nest boxes at Conaway Ranch (16 hectares) as a high-density treatment. We installed 16 nest boxes at Russell Ranch (8 hectares) and 6 nest boxes at Putah Creek (5 hectares) as low-density treatments in from 1998 to 99. We added Roosevelt Ranch as a new study site in 2008. We installed 49 nest boxes in part of Roosevelt Ranch (277 hectares) as a low-density treatment, and 51 nest boxes in the remaining part of Roosevelt Ranch (35 hectares) as a high-density treatment. However, since females moved among treatment areas, we considered Roosevelt Ranch as a single site in the population-level analyses. Accordingly, all densities were established at the start of the current study, although some boxes may have broken or fallen and there is some variation among years as reported in Table 2 and incorporated in the analysis. We searched sites for natural cavities, and evidence of wood duck nests in natural cavities, but found none; hence, we assume that density of nests in boxes approximates the actual nesting density at these sites. Boxes were bolted 1.5–4 m high on an existing tree, within 10 m of water (Russell, Conaway, Putah) or attached to 3 m metal poles with a sliding fixture, within 2–5 m of water (Roosevelt Ranch). Each box was fitted with an antenna circling the box entrance, connected to a custom radio frequency identification (RFID) reader and 12V battery; the RFID readers recorded wood duck females carrying passive integrated transponder (PIT) tags with unique codes whenever they entered or exited the boxes during the breeding season. Logged RFID reads were used to determine onset of incubation and to corroborate the identity of the incubating female as determined by capture on the nest. Box locations were recorded with GPS.

### Field methods.

At the onset of each breeding season (mid-February), boxes were checked weekly for nesting activity: “bowling” of wood shavings inside the box, which indicated that a wood duck female had rearranged them, or the presence of eggs. Once a box showed nesting activity, it was checked approximately every 2 days. New eggs were numbered at each nest check until incubation began; incubation onset was confirmed when nest checks revealed that eggs were warm and covered with a layer of down, from which we estimated likely date of hatch (~30 days from incubation onset, Haramis 1990).

Adult females were captured in nest boxes by closing the box entrance with a wooden plug. At initial capture, females were banded with aluminum USGS numbered bands and injected with a unique PIT tag for permanent identification; mass (to the nearest 5g) and tarsus length (to the nearest mm) were measured at the first and all subsequent captures. Females were generally scheduled for capture near the end of incubation, to prevent nest abandonment associated with possible capture stress; hence size measurements (mass, tarsus) of females were standardized by reproductive stage (i.e., late-incubation) instead of calendar date. A blood sample was taken by pricking a female’s tarsal or alar vein with a 20-gauge or 30-gauge needle and collecting droplets onto a filter strip (Nobutu Blood Filter Strip, Advantec MFS, Japan).

Beginning 2 days before the estimated date of hatch, eggs were checked daily for evidence of tapping or external pipping of the eggshell. Upon hatching, each duckling was injected with a unique PIT tag, underneath the skin between the scapulae. Duckling blood samples were taken by pricking the tarsal vein with a 30-gauge needle and collecting droplets onto a blood filter strip.

Blood samples on filter strips were kept at ambient temperature for several hours until returned to lab, at which point they were transferred to a freezer (0°F) for storage until extraction. DNA from samples was extracted 1–4 mo after collection, using DNeasy Blood and Tissue Kit spin columns (Qiagen, USA), according to manufacturer’s protocol, or using a plate-extraction method (Ali et al. 2016).

### Genotyping and genetic sexing.

We genotyped females and ducklings at 19 microsatellite loci: APH01, APH02, APH08, APH09, APH13, APH18, APH19, APH20, APH23, APH25 (Maak et al. 2000, 2003); APL02, APL23 (Denk et al. 2004); BCAμ5 (Buchholz et al. 1998); CM28, CM35 (Stai and Hughes 2003), SFIμ4 (Fields and Scribner 1997); SMO04, SMO07, SMO10 (Paulus and Tiedemann 2003). GTTTCTT tails were added to reverse primers to prevent split peaks. Duckling sex was determined from the genotype at 2 sex-linked loci. Primers P2/P8 (Griffiths et al. 1998) and 1237L/1272H (Kahn et al. 1998) both amplify an intron in the CHD gene on the Z and W sex chromosomes: females are heterozygous (ZW) and males are homozygous (ZZ). Molecular sex was confirmed for PIT-tagged female ducklings that returned to nest boxes as adults (*n* = 17).

Fluorescently labeled microsatellite and sex-linked primers were multiplexed into 3 25 μl reactions (Thow 2019), each consisting of 2.5 μl PCR buffer [750 mM Tris–HCL pH 8.8, 200 mM (NH_4_)_s_SO_4_, 0.1% Tween 20], 2.5 μl 25 mM MgCl_2_, 2.5 μl dNTPs, 0.5 μl DMSO, 0.2 μl *Taq* polymerase (Denville Choice), 6.8 μl water, 7 μl multiplexed primers, and 3 μl template DNA. PCR reactions consisted of an initial denaturation of 5 min at 95 °C followed by 5 min at 85 °C; then 5 cycles of 1 min denaturation at 95 °C, 30 s annealing at 57 °C, and 30 s elongation at 72 °C; 28 cycles of 45 s at 95 °C, 30 s at 57 °C, 30 s at 72 °C; and ending with 30 min final elongation at 72 °C. PCR products were visualized on an ABI 3730 sequencer and alleles scored using STRand analysis software (www.vgl.ucdavis.edu/informatics/strand.php). Genotyping was performed by the Veterinary Genetics Laboratory at University of California, Davis.

### Maternal assignment.

Genetic assignment of ducklings to females was performed using COLONY 2.0 (Jones and Wang 2010). COLONY 2.0 uses multilocus genotypes and full-pedigree likelihood methods to simultaneously infer parentage and sibships. A pairwise likelihood approach, most commonly CERVUS 3.0 (Kalinowski et al. 2007), is often used to assign parentage in wild populations, but COLONY has been found to make fewer errors in assignment compared to CERVUS for populations with female kin structure and partial sampling of mothers (Thow et al. 2022). Additionally, COLONY can identify genetically unique un-sampled parents and assign offspring to them; this feature is particularly useful for conspecific brood parasitic systems in which eggs may be laid by unsampled females (Thow et al. 2022). With either program, incorrect assignment of offspring from nesting females to other females (i.e. errors that falsely suggest brood parasitism) is rare (Thow et al. 2022).

Separate assignments were conducted for the ducklings hatched in each population in each year. All females captured in nest boxes or by RFID in the current and previous year(s) were included as candidate mothers, excluding only females that were known to have died (i.e. were depredated on the nest or recorded shot through BBL hunter band return records). No adult males were genotyped in this study, so males were not included as candidate fathers. We specified an outbreeding model, with no known sib-ships, no excluded mothers, no excluded sib-ships, and no sib-ship scaling or size prior. We allowed a polygamous mating system for males and females. We conservatively set the probability that a mother is included in the female candidates to 0.7, to allow for high numbers of un-captured, un-sampled exclusive parasite females; setting this parameter lower than the true probability may reduce COLONY’s reported confidence in an individual assignment, but does not change the identity of the assigned parent(s) (Thow et al. 2022). We selected the longest processing run option, using full likelihood approach, with 4 replicates to reduce sampling bias (Wang 2016; Thow et al. 2022). We accepted all maternity assignments made regardless of probability, since errors are not associated with low probabilities (Thow et al. 2022), using the BestCluster output.

### Parasitic ducklings.

Parasitic ducklings were identified by comparison between the genetically assigned mother and the incubating female. Identity of the incubating female for each nest was known from her capture on the nest, or by RFID reads indicating incubation (i.e. full days spent on the nest until the eggs hatched). Ducklings that were genetically assigned to the incubating female were categorized as “nest” ducklings. Ducklings that were genetically assigned to a female other than the one that incubated them, including to unsampled females inferred by COLONY, were categorized as “parasitic” ducklings.

### Female alternative reproductive tactics.

Female ARTs were determined by the hatching location of her ducklings. Females that incubated a nest and were assigned maternity only to ducklings that hatched from that nest were categorized as “Nest” females. Females that incubated a nest and were assigned maternity to ducklings that hatched from that nest *and* ducklings that hatched from a different nest were categorized as “Nesting Parasite” females; because sex allocation may change across the laying sequence (Cassey et al. 2006; Bowers et al. 2014), sex allocation for nest ducklings and parasitic ducklings were considered separately for Nesting Parasite females, which laid both. Females that did not incubate a nest but were assigned maternity to ducklings were categorized as “Parasite” females.

### Statistical analyses.

Following other studies of offspring sex ratio in ducks, in this study, we analyzed secondary sex ratios only, defined as the ratio of male to female ducklings at hatch (Blums and Mednis 1996). A few eggs in some successful clutches did not hatch due to embryonic mortality during incubation, or because their development was not synchronous with the rest of the clutch.

To test predictions from multiple sex allocation hypotheses (Table 1), we analyzed offspring sex allocation at the population and the individual level. At the population level, we used generalized linear models (GLMs) to predict *Z*-score sex ratio (Thogerson et al. 2013) produced for each site-year: 2013–2015 at Putah Creek, 2013–2016 at Russell Ranch, 2014–2016 at Conaway Ranch, and 2015–2016 at Roosevelt Ranch (*n* = 12). Data from Conaway Ranch 2013 were excluded because excessive predation of hens and clutches that year precluded complete genotyping of offspring, and therefore the number and sex of offspring produced. Putah Creek was not monitored in 2016, and complete sampling of offspring was not initiated at Roosevelt Ranch until 2015. For each population predictor variable (e.g. female density), we specified separate models to predict the sex ratio of 3 categories of offspring: all ducklings produced, nest ducklings (i.e., those incubated by the genetically assigned mother), and parasitic ducklings (i.e. those incubated by a female other than the genetically assigned mother). We examined these 3 categories separately to allow patterns of sex allocation to vary by duckling type.

At the individual level, females were often observed over multiple years (*n* = 284 observations of 175 females). Therefore, we used generalized linear mixed models (GLMMs) to fit our data, including female identity as a random effect with a varying intercept. We used the bglmer function (from package blme, Dorie and Dorie 2015) to obtain nonzero random effects for individual females. Models were specified with a binomial error structure and a logit link, with separate models predicting the proportion of males produced by a female each year in 3 categories: proportion of her total offspring that year, of her nest offspring that year, and of her parasitic offspring that year. Effects are reported as beta-coefficients plus standard error; positive beta-coefficients represent an increase in proportion of males produced.

First, we tested predictions from the local resource competition and local resource enhancement hypotheses, that females bias the sex ratio of offspring according to local resource availability. Female density is a common proxy for resource availability, and we defined it in 2 ways. *Females per hectare* were the number of females that bred in a given site in a given year, divided by the site area. *Nearest-neighbor distance* was the shortest Euclidian distance in meters (determined from GPS locations) from the nest box used by the focal female and another occupied box. Average nearest-neighbor distance was the average of the distances to the 2closest occupied boxes for individual females; the average of this value was taken for all individual females to determine the population nearest-neighbor distance. The distribution of average nearest-neighbor distances was right-skewed for individual females across all populations, so this variable was log-transformed in individual models; log-transformation was not necessary for population averages. A positive effect of females per hectare on proportion males produced, or a negative effect of nearest-neighbor distance on proportion males produced, would support the local resource competition hypothesis that wood duck females produce more sons at high density. We used *nest site availability* as another measure of resource competition at the population level, defined as the average number of nest boxes available per breeding female. A negative effect of nest site availability on the proportion males produced would support the local resource competition hypothesis that females produce more sons when nest sites are relatively scarce.

Additionally, since LRC predictions are particularly applicable to competition with relatives (West et al. 2005), we analyzed sex ratios of ducklings produced in populations that varied in average relatedness among females, and by females that differed in their average relatedness to their 2 closest neighbors. We used ML-Relate (Kalinowski et al. 2006) to estimate pairwise relatedness, *r*, between adult females present in the population each year. A positive effect of relatedness on the proportion males produced would support the local resource competition hypothesis that females produce more sons when competition with relatives is high.

Next, we tested predictions from the Trivers–Willard hypothesis that females bias the sex ratio of offspring according to their own structural size or body condition. For these predictions, we fit separate binomial GLMMs of total, nest, and parasite ducklings; size or body condition were tested as fixed effects, and female identity was included in all models as a random effect. We used tarsus length as our estimate of female structural size (Jaatinen et al. 2013). We defined body condition as the residual from a linear model of mass on tarsus length, using the mass at first capture of the year. To determine if variation in female body condition could have contributed to site-specific variation in offspring sex ratio, we fit an ANOVA of condition residuals by the site.

Last, we tested the effect of the 3 alternative reproductive tactics of female wood ducks (nesting, parasite, and nesting parasite) on offspring sex allocation by fitting binomial GLMMs including ART as a categorical fixed effect. We compared (1) the sex ratio of total ducklings produced by all 3 ARTs, (2) the sex ratio of nest ducklings produced by nest vs. nesting parasite females, and (3) the sex ratio of parasitic ducklings produced by parasite vs. nesting parasite females. A higher (male-biased) sex ratio produced by parasite females or a lower (female-biased) sex ratio produced by nesting parasite females would support Silk’s (1983) expansion of the local resource competition hypothesis that females bias sex ratio according to their own competitive ability. To determine if nesting parasite females produced distinct sex ratios according to each of their dual tactics, we fit a binomial GLMM of proportion male ducklings restricting the dataset to nesting parasite females only (*n* = 66 observations of 61 females), with duckling type (“nest” vs. “parasite”) as a fixed effect. A higher (male-biased) sex ratio for parasitic vs nest offspring would highlight laying-order effects in females laying supernumerary eggs. For all of the models in this section, we included site as a fixed effect to control for the effect of the experimental density treatment.

## Ethical note

This research was approved by the Animal Care and Use Committee at the University of California, Davis (protocols #17535, 19281, 22698), California Department of Fish and Game (SC-9565), USFWS (MB230246, MB73393B), and Bird Banding Lab (Master Permit 10562).

## Results

Our nest box treatments were effective in experimentally manipulating female density, generating a range of effective densities comparable to or at the high end of nesting densities of wood ducks in natural cavities (Table 2). However, due to variation in the number of females in each population, the number of boxes per female (i.e., our proxy of nest box competition) did not always reflect female density; for example the highest density site—Conaway Ranch—also had the highest nest box availability. We detected an impact of our manipulation of female density on reproductive behavior, as the proportion of parasitic ducklings increased with fewer boxes per female (R^2^ = 0.34).

Across all years and sites, the population level sex ratio of total ducklings did not differ from parity (*N*_males_ = 1103/2159 total ducklings, 51.09% (48.96–53.22%) male, *P* = 0.322, exact binomial test; Table 2). The majority (82%) of the ducklings produced (i.e., that hatched) during the study were genetically assigned to the female that incubated them, and the rest (18%) were identified as parasitic.

**Table 2.** Number and proportion of male ducklings produced by female wood ducks (*Aix sponsa*) in 4 experimental nest box populations in the Central Valley of California, USA, between 2013 and 2016.

Population	Density treatment	Boxes/ hectare^1^	Average nearest-neighbor distance	Females/ hectare (Effective density)	Boxes/ female (Effective nest competition)	N ducklings (Male, Female)	Proportion male ducklings (95% CI)^2^
Roosevelt Ranch	Low and high	0.32	172–174 m	0.2 (Low)	1.4–1.6 (Medium)	476, 449	51.5% 48.2–54.7% *P* = 0.39
Putah Creek	Low	1.20	46–276 m	1.2–1.4 (Medium)	0.9–1.0 (High)	89, 81	52.4% 44.6–60.1% *P* = 0.60
Russell Ranch	Low	1.48–1.98	63–117 m	0.9–1.4 (Medium)	1.5–1.7 (Medium)	180, 143	55.7% 50.1–61.2% *P* = 0.045
Conaway Ranch	High	3.58–4.30	25–47m	1.7–2.2 (High)	2.0–2.6 (Low)	358, 383	48.3% 44.7–52.0% *P* = 0.38

^1^Several boxes were added at Russell Ranch and Conaway Ranch between the 2014 and 2015 nesting seasons.

^2^Calculated with 2-sided exact binomial test.

### Local resource competition or enhancement

**Figure 1.**
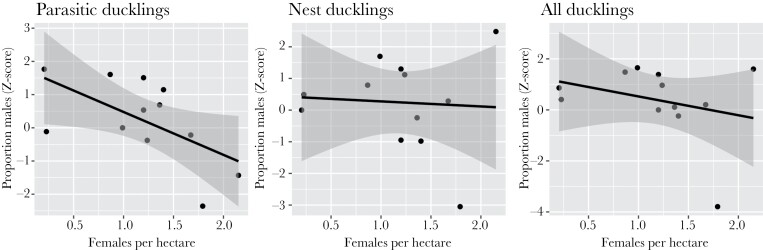
The proportion of males produced as a function of female density for (a) parasitic ducklings, (b) nest ducklings, and (c) all ducklings. Each point represents ducklings produced in one population in 1 year (*n* = 12: 4 years from Russell Ranch, 3 years from Putah Creek, 3 years from Conaway Ranch, and 2 years from Roosevelt Ranch), in the Central Valley of California, USA.

*Density*. At higher population densities, significantly fewer males were produced at the population level when considering parasitic ducklings only (*β* = −1.29 ± 0.55, *z* = −2.33, *P* = 0.04; *r*^2^ = 0.35, Fig. 1a), but not when considering nest ducklings (*β* = −0.16 ± 0.80, *z* = −0.20, *P* = 0.85; Fig. 1b), or all ducklings combined (*β* = −0.74 ± 0.78, *z* = −0.95, *P* = 0.37; Fig. 1c). When we used an alternative measure of density—average nearest-neighbor distance—there was no change in the production of parasitic (*β* = 0.005 ± 0.005, *z* = 1.05 *P* = 0.32), nest (*β* = 0.005 ± 0.006, *z* = 0.88, *P* = 0.40), or all (*β* = 0.007 ± 0.006, *z* = 1.18, *P* = 0.27) male ducklings at the population level. At the individual level, there was no significant change in production of parasitic (*β* = −0.17 ± 0.13, *z* = −1.31, *P* = 0.19), nest (*β* = 0.006 ± 0.055, *z* = 0.103, *P* = 0.92), or all male ducklings (*β* = −0.022 ± 0.05, *z* = −0.44, *P* = 0.66) with site density, or with log-distance to neighboring females (*β*_parasite_ = 0.16 ± 0.12, *z* = 1.29, *P* = 0.20; *β*_nest_ = 0.02 ± 0.04, *z* = 0.53, *P* = 0.60; *β*_all_ = 0.03 ± 0.04, *z* = 0.90, *P* = 0.37).

**Figure 2.**
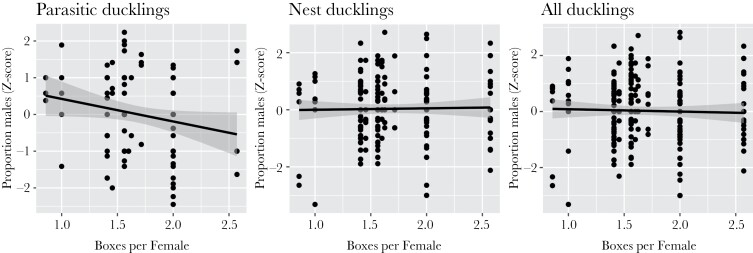
The proportion of males produced as a function of resource availability (i.e. nest boxes per female) for (a) parasitic ducklings, (b) nest ducklings, and (c) all ducklings. Each point represents ducklings produced by a single female in 1 year (*n* = 284) in the Central Valley of California, USA.

*Nest site availability.* Nest site availability ranged from 1.4 to 1.7 boxes/breeding female at the low-density sites (Roosevelt Ranch and Russell Ranch), 0.9–1 box/breeding female at the medium density site (Putah Creek), and 2–2.6 boxes/breeding female at the high density site (Conaway Ranch). Because sites varied in size and in the number of nest boxes installed, female density (females/ha) and nest site availability (boxes/female) were somewhat but not highly related (*r* = 0.12); hence we examined nest site availability (here) separately from female density (above). At the population level, there was no change in production of nest (*β* = 0.29 ± 0.95, *z* = 0.30, *P* = 0.77) or all (*β* = −0.42 ± 0.95, *z* = −0.44, *P* = 0.67) male ducklings with this measure of resource competition. However, there was a trend for fewer parasitic male ducklings to be produced when more boxes were available per breeding female (i.e. with higher resource availability, (*β*_parasite_ = −1.42 ± 0.68, *z* = −2.08, *P* = 0.06; *r*^2^ = 0.30). This pattern was confirmed at the individual level (*β*_parasite_ = −0.61 ± 0.31, *t* = −1.98, *P* = 0.05; *β*_nest_ = 0.05 ± 0.19, *t* = 0.27, *P* = 0.79; *β*_all_ = −0.08 ± 0.19, *t* = −0.45, *P* = 0.65, Fig. 2). Female density explained a higher proportion of the variation in sex ratios of parasitic offspring (*R*^2^ = 35%) than did nest site availability (*R*^2^ = 30%). However, when both variables were included in a linear model, they each had an independent, negative effect on sex ratio, meaning that both contributed to an excess production of females; combined, these 2 variables explained 48% of the variation in the sex ratios of parasitic ducklings (*P* = 0.05).

*Kinship.* Population relatedness (average of estimated relatedness between all pairs of females present during the breeding season) was similar across sites: from 0.04 to 0.06 at the low-density sites (Russell Ranch and Roosevelt Ranch), 0.005 to 0.04 at the medium-density site (Putah Creek), and 0.05 to 0.06 at the high-density site (Conaway Ranch). At the population level, there was no change in production of nest (*β* = 5.58 ± 31.91, *z* = 0.18, *P* = 0.87) or total (*β* = −16.30 ± 31.90, *z* = −0.51, *P* = 0.62) male ducklings with this measure of kinship. However, there was a trend for fewer parasitic male ducklings to be produced at sites with higher female relatedness (*β* = −43.42 ± 23.73, *z* = −1.83, *P* = 0.10; *r*^2^ = 0.25). Individual relatedness (average of estimated relatedness to 2 closest nesting neighbors) ranged from 0 to 0.46 at the low-density sites, 0 to 0.11 at the medium-density site, and 0 to 0.29 at the high-density site. At the individual level, females with a higher average relatedness to neighbors did not produce more male offspring out of parasitic (*β* = −1.47 ± 1.69, *z* = −0.87, *P* = 0.38), nest (*β* = −0.45 ± 0.59, *z* = −0.77, *P* = 0.44), or total (*β* = −0.59 ± 0.55, *z* = −1.07, *P* = 0.29) ducklings.

### Trivers Willard

Structurally larger females did not produce more males when considering parasitic ducklings only (*β* = −0.32 ± 2.58, *z* = −0.13, *P* = 0.90), nest ducklings only (*β* = −0.36 ± 1.05, *z* = −0.35, *P* = 0.73), or total ducklings (*β* = −0.37 ± 0.97, *z* = −0.38, *P* = 0.70). Females in better condition (defined as larger residuals from a linear model of mass on tarsus length) did not produce more males when considering parasitic ducklings only (*β* = −0.08 ± 0.13, *z* = −0.60, *P* = 0.55), nest ducklings only (*β* = 0.02 ± 0.05, *z* = 0.37, *P* = 0.71), or total ducklings (*β*=0.008 ± 0.042, *z* = 0.19, *P* = 0.85). Female condition at the medium- and low-density sites was not significantly different from female condition at the high-density site (*β*_Putah_ = −10.63 ± 13.89, *z* = −0.77; *β*_Russell_ = 4.59 ± 10.33, *z* = 0.44; *β*_Roosevelt_ = 4.60 ± 6.67, *z* = 0.69).

### Female alternative reproductive tactic

**Figure 3.**
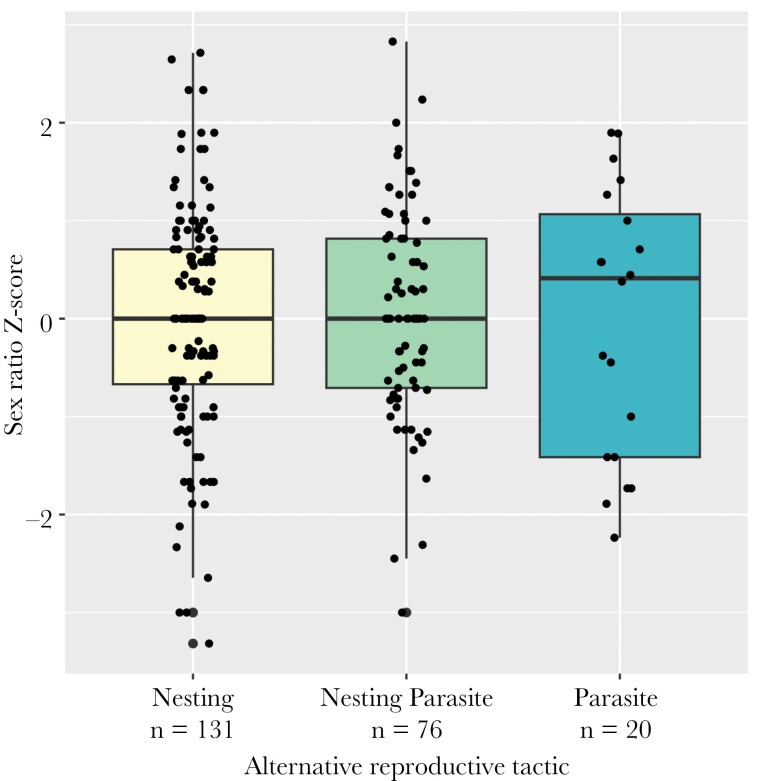
Proportion total male ducklings produced by females using each of 3 alternative reproductive tactics: Nesting female (laid only in the nest she incubated), nesting parasite (laid in the nest she incubated *and* in nests incubated by other females), and parasite female (laid only in nests incubated by other females—did not incubate a nest herself). A *Z*-score of 0 indicates an even sex ratio, consistent with the binomial expectation for a given sample size; positive values represent increasingly male-biased offspring, and negative values represent increasingly female-biased offspring.

**Figure 4.**
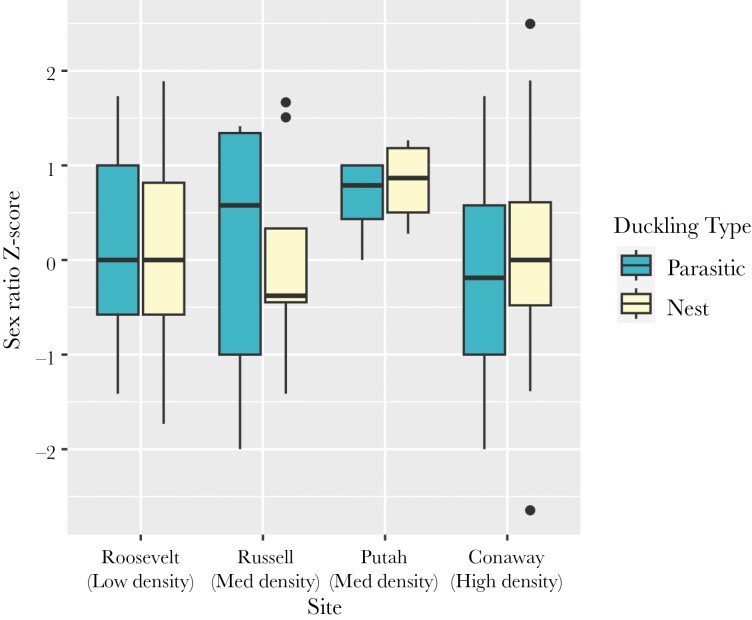
Sex ratios of ducklings hatched in other nests (parasitic ducklings, left boxplots) vs. own nest (nest ducklings, right boxplots) for nesting parasite females at 4 sites across a range of female density. A *Z*-score of 0 indicates an even sex ratio, consistent with the binomial expectation for a given sample size; positive values represent increasingly male-biased offspring, and negative values represent increasingly female-biased offspring.

The sex ratio of total ducklings produced did not vary with the female alternative reproductive tactic (Fig. 3). Specifically, neither parasite females (*β*_P_ = 0.05 ± 0.17, *z* = 0.27, *P* = 0.79) nor nesting parasite females (*β*_NP_ = 0.01 ± 0.08, *z* = 0.19, *P* = 0.85) produced more male ducklings than nesting females. The sex ratio of nest ducklings also did not vary with female ART: nesting parasite females did not produce a different sex ratio of ducklings in their own nests than did nesting females (*β*_NP_ = 0.04 ± 0.09, *z* = 0.40, p = 0.69). Additionally, the sex ratio of parasitic ducklings produced did not vary with female ART: Parasite females did not produce a higher (male-biased) or lower (female-biased) sex ratio of parasitic ducklings than did nesting parasite females (*β*_P_ = 0.10 ± 0.20, *z* = 0.52, *P* = 0.61). Finally, within females, nesting parasite females did not produce a higher proportion of male parasitic ducklings than they did nest ducklings (*β*_parasitic_ = −0.07 ± 0.15, *z* = −0.45, *P* = 0.66; Fig. 4).

## Discussion

In species with strong female–female competition over limited nest sites, such as cavity nesting waterfowl, several theories of sex allocation predict biased offspring sex ratios. However, specific predictions about the direction of effects, at both population and individual level, have not been developed previously for species in which there is strong female natal philopatry and conspecific brood parasitism. Such species offer novel and intriguing systems with which to explore when—or if—biased offspring sex ratios might be expected. As illustrated in our conceptual framework, the predictions are nuanced and could depend on a variety of factors, including nest site availability, female density, female quality or body condition, and kinship. These factors may act in concert, or antagonistically, to produce a variety of outcomes (Table 1).

We tested these predictions using a data set comprising large numbers of nests and females followed over multiple years at 4 sites, large numbers of offspring of known genetic maternity, and experimental manipulations of nest box and female density. Yet, despite the large sample sizes, experimental manipulations, and multiple sites, we failed to find strong evidence that female wood ducks altered offspring sex ratios according to local resource availability (Clark 1978; Emlen et al. 1986), their own body condition (Trivers and Willard 1973), or alternative reproductive tactic (this study).

Why not? We can envision several reasons we did not observe the predicted sex ratio biases. First, we may not have been able to detect sex ratio bias at the individual level—the level at which sex ratio biasing must occur—because of the large clutches wood duck females lay. It has been suggested that females may only be able to bias the sex of their first egg, through selective resorption of oocytes of the nonpreferred sex, without costly “skips” in egg production (Emlen 1997). Hence sex ratio bias should be detected most strongly in single-egg clutches (e.g., Heinsohn et al. 1997; Komdeur et al. 1997), and less so in multi-egg clutches (Emlen 1997). Indeed, across a range of bird species sex ratios for the first egg in a clutch have been more biased than subsequent eggs (e.g., Dijkstra et al. 1990; Vedder et al. 2013; Tschumi et al. 2019). We did not have egg laying dates for individual ducklings in our study, and so we were unable to restrict our analysis to first-laid eggs only. This would be an interesting follow-up for a future study.

Second, females may not have experienced sufficiently high levels of competition for resources to warrant changes in sex allocation. Although we did manipulate female density with our experimental nest box treatment, densities may not have been high enough to generate intense competition for resources. It is possible that all of our study sites, even those at high density, were able to support more adult females than were present. Supporting this view is the fact that at all sites, boxes were still available for nesting. However, our high densities of 1.7–2.2 females/ha are in the upper range of those observed in natural cavities (see Methods). Moreover, an earlier study of cavity-nesting great tits (*Parus major*) found significant changes to sex allocation at densities comparable to ours, and well below saturation (Song et al. 2016).

Third, the costs of parasitism may be sufficiently low in precocial birds that parasitism fails to exert a strong selective pressure in the context of LRC. Empirical analyses of the cost of parasitism are limited in conspecific brood parasites with precocial young, but include reductions in clutch size (e.g. Andersson and Eriksson 1982; Nielsen et al. 2006; Waldeck et al. 2011), reduced hatching success (e.g. Morse and Wight 1969; Lank et al. 1990; Semel and Sherman 2001; Craik et al. 2018), and total nest failure (e.g. Nielsen et al. 2006; Jaatinen et al. 2009). However, these impacts are not seen in all species and are predominantly observed when the frequency of CBP is high (e.g. Andersson and Eriksson 1982; Eadie et al. 1998; Nielsen et al. 2006).

The costs of parasitism might be ameliorated further by kinship among hosts and parasites. Females are the philopatric sex in waterfowl and hosts and parasites might be related (Andersson 2001, 2017; Andersson et al. 2019). Even if parasitism has some level of cost, acceptance of eggs from a female relative—who might not be able to reproduce otherwise—could enhance the inclusive fitness of the host. This would not only reduce the cost of parasitism but contrary to LRC predictions, could favor local resource enhancement (LRE). Although we did not find compelling or consistent evidence of sex ratio adjustment among females in response to local resource availability or their own body condition, the one pattern that did emerge was that significantly fewer males were produced at the population level when considering parasitic ducklings only, and there was a trend for fewer parasitic male ducklings to be produced when more boxes were available per breeding female (i.e. higher resource availability). We consider this further below.

Fourth, it may be that biasing offspring sex does not pay relative to other forms of investment. As in many ducks, wood duck duckling mortality is high (Davis et al. 2009), female recruitment is low (Hepp et al. 1989), and increased maternal investment in egg volume and duckling mass increases duckling survival (Sedinger et al. 2018). Hence, differential investment in duckling size or duckling quality may be more profitable than manipulating offspring sex.

Last and possibly most likely, it may be that biasing investment does not pay at all in this species, as an equal sex ratio is expected if potential payoffs from each sex are equivalent (Charnov 1982). The classic Trivers–Willard hypothesis assumes that one sex (i.e. males in a polygynous species) will benefit more from the maternal condition, but this may not always be the case. Wood ducks are among the most sexually dimorphic of any species of North American waterfowl and the elaborate plumage patterns and displays of males suggest that there is strong sexual selection acting on males for competition for mates (Bellrose and Holm 1994; Semel and Sherman 2001). Conversely, there are many reports of the intense competition among females for nest sites, involving extended fights, occasionally resulting in death (Bellrose and Holm 1994; Harvey et al. 2021). In female ducks, variation in plumage traits has been linked with individual quality and reproductive success (Ruusila et al. 2001a). Female wood ducks have distinct plumages, including eye rings that vary with age and potential dominance (Dooley 2019; Graves and Eadie 2020, Cook 2022), suggesting that social selection acts strongly on females as well. In species with strong male-male competition for mates *and* strong female-female competition for nests, such as wood ducks, sons and daughters may yield equal fitness and hence equal sex ratios. This is in contrast to previous research in cavity-nesting species with male philopatry (e.g. Hjernquist et al. 2009; Song et al. 2016), in which male–male competition for nest sites may magnify variance in male–male competition for mates, generating stronger selection for biased offspring sex ratios than in female-philopatric species.

One potential exception to our null results was the sex ratio of parasitic ducklings, which was female-biased at sites with highest female density, highest nest site availability, and possibly at sites with highest population-level relatedness among females. Because we evaluated the secondary sex ratio (i.e. the sex ratio at hatch), we cannot evaluate the mechanism of this bias; it could arise either from an even sex ratio at laying with differential mortality of male embryos during incubation or alternatively from a bias at laying. Most parasitic ducklings were laid by Nesting Parasite females (data not shown; Thow 2019), who often (but not always) lay parasitically before incubating their own nests (Semel and Sherman 2001, Andersson and Åhlund 2012). Earlier-laid eggs may show a relatively stronger sex ratio bias than later-laid eggs for the reasons outlined above (Emlen 1997), and this may underlie the signal in our data. Our within-female comparisons of Nesting Parasite offspring did not show differences in sex ratios for those laid parasitically versus within her own nest, yet our sample size was not large enough to test for an effect of density, which appeared to drive this pattern. If the female-bias in parasitic ducklings is credible, why should it be? If female ducklings imprint on their natal box, perhaps parasitic ducklings could gain an advantage in nesting there if they recruit, whereas nest ducklings would only be competing with their mother (Pöysä et al. 1997; Ruusila et al. 2001b; but see Weatherhead 1998). Notably for wood ducks, though sites with highest female density and highest nest site availability seem opposed in predictions from local resource competition (reflecting increasing and decreasing competition, respectively), high density of nesting females does *increase* nest site availability for parasitic females; perhaps for a conspecific brood parasite these effects are actually telling us the same thing, that female wood ducks are producing more daughters when local nesting opportunities for those daughters are high.

While our study found limited evidence of sex-ratio bias in offspring of wood ducks, we note that our results may be specific to these populations or this species; similar analyses should be repeated for other species—especially cavity-nesting waterfowl or species with strong female-female competition over other resources such as brood rearing sites. We have also offered a conceptual framework to guide future work, especially when the predictions at an individual and population level might be nuanced and vary. Our framework helps to extend predictions from several widely cited theories of sex ratio allocation to species with strong female philopatry and in which females employ alternative reproductive tactics. The unique features of conspecific brood parasitism bring considerable richness and opportunity to expand the scope for further tests and refinement of sex allocation theory.

## Data Availability

Analyses reported in this article can be reproduced using the data provided by Wells et al. (2024).
